# Dietary stability in ancient Serbia: Isotopic analysis of two middle bronze age Moriš Cemeteries

**DOI:** 10.1371/journal.pone.0344463

**Published:** 2026-04-01

**Authors:** Iride Tomažič, Katherine Pompeani, Kara Larson, Amy Nicodemus, John O’Shea, Lidija Milašinović, Alicia Ventresca-Miller

**Affiliations:** 1 University of Michigan Museum of Anthropological Archaeology, Ann Arbor, Michigan, United States; 2 University of Michigan Department of Anthropology, Ann Arbor, Michigan, United States; 3 University of Pittsburgh, Pittsburgh, Pennsylvania, United States; 4 University of Wisconsin La Crosse, La Crosse, Wisconsin, United States; 5 National Museum of Kikinda, Kikinda, Serbia; University of Padova: Universita degli Studi di Padova, ITALY

## Abstract

Food stability refers to a state of consistent and reliable access to key dietary resources and is a crucial factor in the resilience and growth of communities throughout history. The study of human diets has been a focus of archaeological research over recent decades. Isotopic analyses provide unique insight into the breadth and evolution of food consumption, often reflecting broader environmental and social shifts while also indicating human resilience and adaptability to various stressors. Rarely, however, are we able to observe subsistence economies over extended periods within the same archaeologically defined cultural group. This research is the first isotopic project on Bronze Age diet of the Moriš culture (roughly 2700−1500 BCE), and one of the few isotopic studies in the Carpathian Basin. This research presents stable carbon and nitrogen isotopic data from human and animal bone collagen recovered at four Moriš sites. This includes two cemeteries (Mokrin and Ostojićevo) and two settlements (Kiszombor Új Élet and Klárafalva Hajdova), all located within the southern Carpathian Basin. Isotopic analysis of human collagen reveals minimal variation among individuals buried in the cemeteries over the span of 550 years (2100−1550 BCE). Overall, there was food stability during the Early and Late Moriš, with only a slight change in diet towards the end of the Late Moriš period.

## Introduction

Food stability relates to the concept that there is consistent and nutritious food that meets dietary needs (i.e., macronutrients and micronutrients) that are essential for the maintenance and growth of human populations [1]). Archaeologists have long argued that food stability and security can be identified via studies of access, availability, preference, and/or scarcity [2,3]. One way to clarify changes, or stability, in availability of foods is to provide evidence for dietary intake over long time spans. In this study, we examine human dietary intake via stable isotope analysis focusing on dietary patterns among Bronze Age Moriš communities (2100−1550 BCE) over a 550-year time span at two cemeteries. This period of time is marked by changes in social organization and economic activity within the larger Moriš cultural group as evidenced by shifts in the material culture recovered from household and mortuary contexts, alongside evidence for an intensification in metallurgical production and domestic horse breeding.

Categorized as an Early and Middle Bronze Age archaeological complex within the southeastern Carpathian Basin, the Moriš culture extends over a 1200-year period and has been extensively studied [4–23]. Authors generally agree that there are changes in material culture assemblages (e.g., mortuary offerings, ceramic styles) over the 1200 years of Moriš existence as a distinct archaeological entity which can be broadly divided into two periods: Early Moriš (2500–1850 cal BCE) and Late Moriš (1850–1500 cal BCE) [11]. These periods are separated according to changes in material culture, with the Late Moriš characterized by an intensification of metal production and long-distance trade, along with an increased emphasis on horse breeding after 1850 cal BCE [24:605–606].

This paper analyzes isotopic data from human remains and faunal assemblages representing both Early and Late Moriš, from both settlement and cemetery contexts. While the settlement of Kiszombor-Új-Élet dates to the Early Moriš period, the settlement site of Klárafalva Hajdova and the Mokrin cemetery fall at the transition from the Early to the Late Moriš periods. The cemetery of Ostojićevo falls partially in the Early Moriš, but primarily within the Late Moriš period. Radiocarbon dating conducted by O’Shea et al. [24] initially identified a gap in interment at the Ostojićevo cemetery between 1800 and 1600 BCE. However, new research by Milašinović et al. (2024) indicates that while shifts in mortuary treatment occurred after 1850 BCE, the cemetery itself appears to have been continuously utilized throughout the entire Moriš period [25]. There are clear shifts in material culture evident between the Early and Late Moriš. For example, at the Late Moriš cemetery at Ostojićevo, there is a shift in funerary practice (after 1850 BCE) towards a decrease in the presence of grave goods, including very few metal artifacts. Additionally, this later phase of the cemetery is marked by an unusually large number (approximately 70%) of infants and young children (n = 142) buried in ceramic vessels [26].

**Fig 1:**
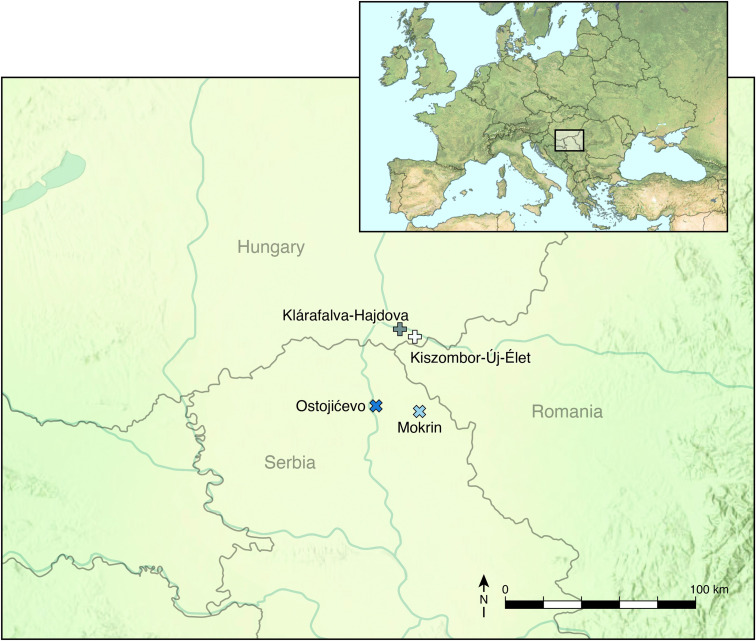
Map of the Archaeological Sites mentioned in the text.

**Fig 2:**
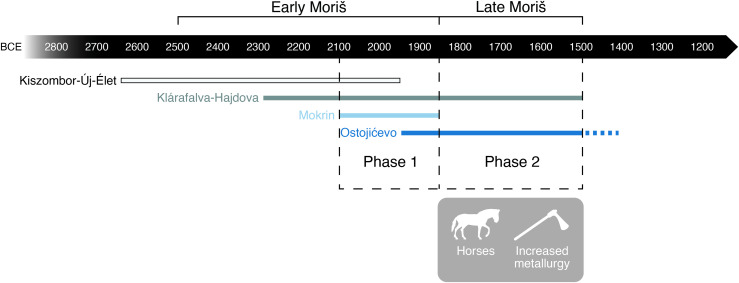
Sites organized chronologically into Early and Late Moriš Stages (as well as phases of cemetery use).

Archaeologically documented changes in material culture are sometimes accompanied by corollary shifts in patterns of subsistence or dietary intake. Our work investigates this premise vis-à-vis assessing whether food stability, or uncertainty, characterized the transition from the Early to the Late Moriš period. Thus, in this paper, we present the stable carbon and nitrogen isotope results from human (n = 78) and faunal (n = 23) remains from two Moriš cemeteries — Ostojićevo and Mokrin, alongside two Moriš settlements (Kiszombor-Új-Élet and Klárafalva-Hajdova) (Fig 1). We analyzed the data based on temporal differences (Fig 2) split into early (1) and late (2) phases (Fig 2). Phase 1 corresponds to the transition periods from the Early to Late Moriš, with data from Mokrin, Kiszombor-Új-Élet, Klárafalva Hajdova, and the early phase of Ostojićevo (dating until 1850 BCE). Similar mortuary treatments were represented at both Mokrin and during phase 1 of Ostojićevo. Phase 2, in contrast, is represented by Ostojićevo from 1850 BCE until the end of the Moriš cultural complex around 1500 BCE. While communities no longer buried their dead at Mokrin during Phase 2, inhumations continued at the cemetery at Ostojićevo [24,25].

## Background

The Moriš (also called Maros/Mureş) archaeological complex, located in the southeastern Carpathian Basin (e.g., present-day Hungary, Serbia, and Romania), represents a network of interconnected communities that lived during the Early and Middle Bronze Ages (roughly 2700–1500 BCE) [24]. While through time the archaeological complex changed names from Perjamos (after the type site Periam which was discovered in the early 1900’s) [20] to Moriš [13], it was via the work of V.G. Childe that the cultural complex became widely known [4]. Since V.G. Childe wrote about the Moriš [4], numerous settlements and cemeteries have been systematically investigated [4–23].

Previous research suggests that throughout their 1200-year evidence of habitation in the region, Moriš communities shared cultural similarities, including lifeways and burial practices. The Moriš culture area was largely characterized by seasonal to permanent freshwater wetlands. Communities inhabited the region along the Moriš River, the eastern bank of the Tisza River, and areas north of the Danube [9,27]. Settlements, usually placed on terraces above the wetlands, included densely occupied areas with rectangular houses constructed out of wattle and daub, with thatched roofs [28:358], and usually surrounded by ditches. While the purpose of the ditches is still unresolved [8], they may have been used for defense, separation of space, or to manage seasonal flooding. Demographic expansion also seems to have occurred in the Late Moriš period, sometime after 1850 BCE, as evidenced by a greater number of settlements.

Seasonal wetland environments placed natural restrictions on settlement locations in the Moriš culture area [9] and, in some instances, the timing of activities. Data from settlements indicates that the Moriš engaged in a vast range of craft production tied to local and regional economies, including ceramic production, metallurgy, textiles, and long-distance trade [4,8,11]. Moriš communities utilized a variety of subsistence and economic practices, generally sustaining themselves through a combination of agriculture and animal herding [11]. Zooarchaeological and paleobotanical evidence from the settlement sites of Kiszombor Új Élet, Klárafalva Hajdova, and Pecica Şanţul Mare, for example, show a vast array of domesticated plants, including barley and einkorn wheat, legumes, peas, and lentils [9,29]. Individual seeds of millet were also recovered from Pecica Şanţul Mare, Kiszombor Új Élet, and Klárafalva Hajdova (Jones, Hannon and Hunter, forthcoming). However, none of these seeds have been dated [29:114] and current evidence suggests that millet was not introduced until the Middle Bronze Age (1900–1500 BCE) [30].

The high salinity of the seasonal wetland soils surrounding many Moriš settlements was ideal for grazing during dry periods [10,27,31,32]. The Moriš herded cattle (*Bos taurus*), sheep (*Ovis aries*), goats (*Capra hircus*), and horses (*Equus caballus*), and also kept pigs (S*us scrofa*). Occasional remains of dogs (*Canis familiaris*) have also been identified [11,33]. However, the percentage of herd species varied temporally and from site to site [11]. For example, Nicodemus suggests that Pecica Şanţul Mare specialized in horse breeding primarily during the peak of its habitation (around 1800−1700 BCE) [11]. Hunting and gathering activities were also part of Moriš subsistence practices, especially at sites closer to the Tisza, such as Klárafalva Hajdova, where evidence of fishing carp and pike was found [9,11], although it is unclear the extent to which the Moriš relied on wild-caught versus domestic resources.

In addition to their subsistence activities, the social structure of the Moriš communities is reflected in their burial practices. Moriš cemeteries were most likely utilized by multiple surrounding communities and were located separately from settlements. While it remains unclear which settlements utilized the cemeteries, it is possible that several groups utilized the cemeteries simultaneously. The presence of exotic trade goods, along with pottery, stone tools, weapons, faience beads, animal grave goods (including pierced canines), and metal artifacts such as sashes, dress pins, headdresses, and weapons, offers insight into trade and interaction, craftsmanship, social status, and daily life of Moriš communities.

Work by Žegarac et al. [17] conducted on the Mokrin cemetery shows that individuals were buried according to family and kinship ties. Despite occasional burials with multiple individuals, burials were primarily single inhumations. Similarly, many burials presented elaborate assemblages of grave goods that followed strict mortuary rules. The majority of the deceased were buried in a flexed body position, oriented north-south, their face facing east, while a smaller portion was oriented east-west, face pointing north or south [8]. The grave inventories varied according to osteologically estimated sex, and age, and likely reflect intra-community variation in gender expression tied to age or social differences such as status [8]. Most males were buried on the left side, head pointing north. The majority of females, on the other hand, were buried on their right side, head pointing south [8,15–17]. Children under three years of age are absent from Morkin and most other Moriš cemeteries. These patterns are largely replicated at Ostojićevo, although most females were buried with their heads to the south or west and males to the north or east, while children represent the majority of all interred individuals at Ostojićevo [15,16,26].

### Site description and chronology

Ostojićevo cemetery is located 40 km south of the Tisza-Moriš River confluence and 20 km west of Mokrin cemetery. Excavated from 1981 to 1991, Ostojićevo cemetery contains 285 graves. Investigations at Mokrin were conducted from 1958 to 1965 and uncovered more than 312 graves. However, the excavation at the Mokrin cemetery recently indicated that an estimated 50−100 graves remain undisturbed [17]. While Mokrin cemetery dates from 2100−1850 cal BCE [34], Ostojićevo dates from 1950−1550 BCE. According to mortuary treatments, there appear to be at least two phases of the cemetery of Ostojićevo, with the early phase dating from 1950−1850 BCE and a later phase post 1850 BCE. The earlier phase is characterized by a greater amount and diversity of grave goods [8,13,25], with both the quantity and diversity of grave offerings decreasing in the later period [25]. Additionally, the later phase of the cemetery is marked by an immense increase in the number of burials of neonates and infants, the vast majority of whom were buried in ceramic vessels, with children accounting for approximately 70% of the total number of burials at Ostojićevo.

The use phases of the respective cemeteries partially overlap with occupation of the settlement of Klárafalva Hajdova, a tell site dating from 2300 to 1500 cal BCE. Briefly, Klárafalva-Hajdova is a 1.4-ha fortified tell site situated within a low, wooded floodplain and presents compelling evidence for fishing [24,35,36]. Areas adjacent to the settlement consisted of seasonal or permanent wetlands with significant gallery forests along the river [11]. Despite its strategic location on a significant regional waterway, the inhabitants would have had limited access to year-round dry, arable land, though the southernmost portion of the site’s catchment is bounded by a loess terrace with an open steppe forest [8,11].

Kiszombor-Új-Élet (2700−1950 cal BCE) is a 5.8 ha, stratified site located ca. 9 km southeast of Klárafalva-Hajdova and situated within a 1-1.5 km strip of dry steppe forest areas adjacent to the Porgány Creek. Located further away from the Moriš River, it has comparatively less evidence of fishing [8,28,29,37]. The greater availability of flood-free land at Kiszombor-Új-Élet supported a larger, more open settlement with lower occupation density and greater house spacing compared to Klárafalva-Hajdova [11,28]. The settlement is bound by steppe forests with highly productive loess soils that are well-suited to agriculture and herding, with marshes and wet meadows also present neaby.

The settlements of Klárafalva Hajdova and Kiszombor Új-Élet present a well-established chronology and extensive analysis of faunal material. Although located at a distance from the two cemeteries, they were utilized as proxies for developing a temporally constrained faunal baseline. Situated within the Moriš River catchment, these settlements represent fortified, high-density areas, offering valuable insights into ecological variations in animal husbandry across the Moriš region (*i.e.*, riverine, freshwater marsh, gallery forest, and open grassland). To refine the data further, animal remains recovered adjacent to human burials at the Ostojićevo cemetery provide the closest comparative evidence for individuals and enhance our understanding of localized environmental conditions.

## Materials and methods

### Osteological analyses

Estimation of age-at-death and assessment of osteological sex are documented in Rega [38] for Mokrin and Pompeani [16] for Ostojićevo. Briefly, estimation of adult age-at-death at Ostojićevo and Mokrin were based on documenting and seriating age-related changes in the auricular surface [39] and/or pubic symphysis [40], with comparable methods used for both cemeteries. When present, union of late-fusing epiphyses (e.g., medial clavicle, iliac crest, humeral head) were used to confirm or adjust ages among young adults in the Ostojićevo skeletal assemblage. Rather than assign a point age estimate, Ostojićevo individuals were assigned to one of eight adult age categories based on: (1) utility in conducting comparative demographic analysis, especially with published age-at-death data collected by Rega [38] from Mokrin; (2) presence and appearance of developmental landmarks; and (3) reflect methodological limitations of greater uncertainty with increased age. Several intermediate categories (i.e., Juvenile II/Adult I, Adult I/II, and Adult III/IV) were developed to account for individuals with conflicting or ambiguous evidence for age-at-death. Finally, an Adult (A) category was used for individuals with insufficient preservation for determination of age-at-death, but who exhibited complete fusion of extant epiphyses indicating an age greater than approximately 22 years. Adult age-at-death categories for both Mokrin and Ostojićevo include: Adult I (20–30 years), Adult II (>30–40 years), Adult III (>40–50 years), Adult IV (>50 years), and Adult A (>22 years).

Subadult age-at-death at Ostojićevo was assessed based on dental development [41–45] and epiphyseal and apophyseal fusion [46‒46–48], with the sample divided into eight age categories: neonate (<1 year), infant I (1–3 years), infant II (3–6 years), child I (6–9 years), child II (9–12 years), juvenile I (12–15 years), juvenile II (>15–20 years), and subadult Indeterminate (0–18 years). Subadult age classes and intervals are adapted from Buikstra and Ubelaker [49] to reflect uncertainty in age estimation from macroscopic analysis of subadult remains as it relates to stages of postnatal human growth and development. Subadult age classes for the Ostojićevo sample are broadly comparable with those used by Rega [38] for Mokrin.

Biological sex was estimated for individuals greater than 20 years-at-death. Estimation of biological sex was based on nonmetric macroscopic analysis of the skull, mandible, and ossa coxae according to criteria outlined in Buikstra and Ubelaker [49] and Acsádi and Nemeskéri [50]. When present, the Phenice [51] criteria were applied to assessing sexual dimorphism in the pubic and subpubic region, specifically characteristics of the ventral arc, subpubic angle, and ischiopubic ramus. A general description of sexually dimorphic traits was recorded for each adult individual. Following analysis, each adult skeleton was assigned to one of five categories that reflect variation in morphology and preservation: M (male), MI (probable male), FI (probable female), F (female), or I (indeterminate adult) [16]. Subadult biological sex was not measured due to issues of preservation and methodological inaccuracies [52].

### Stable carbon and nitrogen isotopes

Stable isotope analysis of bone collagen has been widely used to reconstruct human and animal diets [53–57]. δ^15^N_collagen_ follows a stepwise pattern of trophic level isotopic enrichment ranging from 3-5‰ depending on factors such as manuring, temperature, aridity, protein quality, and physiological stress [58–60]. In contrast, δ^13^C_collagen_ shows a moderate trophic level effect of 0–2‰, with natural variation in carbon isotopic signatures variously linked to temperature, altitude, and photosynthetic pathway [60,61,62]. The relationship between the δ^13^C of the diet and the δ^13^C of bone collagen is more complex than for nitrogen, as non-protein carbon in the diet can be incorporated in body protein (*e.g.*, greater synthesis of non-essential amino acids in a low-protein diet) [63,64]. Controlled-feeding studies have found that bone collagen δ^15^N and δ^13^C reflect the protein portion of the diet in mammals, with routing of dietary amino acid >50% for collagen carbon and ca. 72% for collagen nitrogen [65,66]. Thus, nitrogen values are more representative of the protein component of diet than carbon.

Stable isotope analysis of human bone collagen was conducted to examine dietary patterns at the Bronze Age cemeteries of Ostojićevo (ca. 1950–1550 BCE) and Mokrin (ca. 2100–1850 BCE). All human samples were obtained with permission from the Narodni Muzej Kikinda, Serbia. No permits were required for the described study, which complied with all relevant regulations. Human bone samples from Ostojićevo (*n* = 54) and Mokrin (*n* = 24) were collected from individuals >18 years at death. Ribs were preferred as they have a faster cortical turnover rate (ca. 4% per year) than other skeletal elements (*i.e.*, 1.5-3.0% per year after age 20 for the femur) and thus reflect diet during a shorter period prior to death [57,67–69]. However, due to differences in excavation strategies that influenced which skeletal elements were retained, ribs were unavailable from Mokrin. Thus, most samples from Mokrin were taken from long bone shafts (see Table 1), which corresponds to a period of up to several decades prior to death, especially in older adults [57].

**Table 1:** Stable carbon and nitrogen isotope values among human individuals at Ostojićevo and Mokrin (the asterisk * denotes samples that were not used in analysis).

Site	Grave	Sample	Age	Sex	%C	δ13C	% N	δ15N	C:N	Phase	
Ostojićevo	Grave 100, Trench 28	Human rib	younger adult II	male	25.07	−19.46	9.19	10.32	3.2	2	*
Ostojićevo	Grave 105, Trench 31	Human rib	infant IA	subadult	42.05	−19.23	15.97	11.94	3.1	1	
Ostojićevo	Grave 106, Trench 31	Human rib	younger adult I	male	37.13	−20.17	14.35	12.83	2.9	1	
Ostojićevo	Grave 107, Trench 31	Human rib	younger adult II	male	41.58	−19.25	15.92	11.06	3.0	2	
Ostojićevo	Grave 114, Trench 32	Human rib	younger adult II	female	40.29	−19.00	15.26	10.81	3.1	1	
Ostojićevo	Grave 115, Trench 34	Human rib	older adult	female	39.10	−19.19	14.95	11.17	3.1	1	
Ostojićevo	Grave 116, Trench 34	Human rib	child II	subadult	26.81	−20.02	10.20	9.65	3.1	1	*
Ostojićevo	Grave 119, Trench 37	Human rib	child I	subadult	37.33	−19.12	14.08	8.98	3.1	1	
Ostojićevo	Grave 12, Trench 7	Human rib	neonate	subadult	40.86	−19.88	14.75	11.75	3.2	2	
Ostojićevo	Grave 120, Trench 37	Human rib	younger adult II	female	19.43	−19.20	7.16	11.44	3.2	1	*
Ostojićevo	Grave 121, Trench 37	Human rib	adult	male	24.00	−19.72	9.02	10.09	3.1	1	*
Ostojićevo	Grave 127, Trench 39	Human rib	juvenile A	subadult	29.95	−19.25	11.43	11.81	3.1	1	
Ostojićevo	Grave 13, Trench 7	Human fibula	infant IA	subadult	41.40	−19.32	15.19	14.90	3.2	2	
Ostojićevo	Grave 133A, Tr 40	Human rib	neonate	subadult	39.49	−19.40	15.56	10.76	3.0	2	
Ostojićevo	Grave 133B, Tr 40	Human rib	neonate	subadult	32.29	−20.12	12.77	14.37	3.0	2	
Ostojićevo	Grave 135, Trench 40	Human rib	Child I	subadult	40.98	−20.91	15.79	11.20	3.0	1	
Ostojićevo	Grave 15, Trench 8	Human rib	neonate	subadult	39.78	−19.59	14.57	12.54	3.2	2	
Ostojićevo	Grave 150, Trench 54	Human rib	neonate	subadult	38.84	−20.46	14.61	15.15	3.1	2	
Ostojićevo	Grave 153, Trench 56	Human rib	younger adult I	female	23.32	−19.66	8.74	11.54	3.0	2	*
Ostojićevo	Grave 155, Tr 49	Human rib	mature adult	male	42.91	−17.21	16.53	11.99	3.0	2	
Ostojićevo	Grave 156, Tr 49	Human rib	younger adult II	male	30.60	−19.29	11.60	11.42	3.1	1	
Ostojićevo	Grave 157, Tr 58	Human rib	infant IB	subadult	27.70	−19.42	10.53	10.28	3.1	2	
Ostojićevo	Grave 160, Tr 50	Human rib	Child I	subadult	38.27	−19.74	14.93	10.91	3.0	2	
Ostojićevo	Grave 165, Tr 59	Human rib	Child II	subadult	7.54	−19.68	2.38	10.74	3.7	2	*
Ostojićevo	Grave 173, Tr 62	Human femur	infant IB	subadult	39.67	−19.54	15.06	11.60	3.1	2	
Ostojićevo	Grave 176, Tr 60	Human rib	younger adult I	female	32.93	−19.30	12.43	10.69	3.1	2	
Ostojićevo	Grave 18, Trench 9	Human rib	child I	subadult	41.83	−19.48	15.39	11.45	3.2	2	
Ostojićevo	Grave 183, Tr 70	Human rib	infant IA	subadult	40.18	−19.72	15.59	14.51	3.0	2	
Ostojićevo	Grave 191, Tr 71	Human rib	mature adult	female	39.71	−19.78	15.38	11.04	3.0	2	
Ostojićevo	Grave 199, Tr 67	Human rib	mature adult	male	35.16	−19.79	13.54	11.18	3.0	2	
Ostojićevo	Grave 20, Trench 10	Human rib	neonate	subadult	40.50	−19.50	14.94	12.57	3.2	2	
Ostojićevo	Grave 203, Tr 77	Human rib	older adult	male	14.09	−19.46	4.95	10.89	3.3	1	*
Ostojićevo	Grave 205, Tr 77	Human rib	neonate	subadult	39.86	−19.14	15.35	13.11	3.0	2	
Ostojićevo	Grave 207, Tr 83	Human rib	infant IA	subadult	39.88	−19.14	15.55	12.29	3.0	2	
Ostojićevo	Grave 208, Tr 83	Human rib	adult	female	41.63	−19.71	16.34	9.62	3.0	2	
Ostojićevo	Grave 209, Tr 83	Human rib	child II	subadult	41.44	−18.47	16.36	10.96	3.0	2	
Ostojićevo	Grave 21, Trench 10	Human rib	mature adult	female	41.17	−19.58	15.13	10.87	3.2	2	
Ostojićevo	Grave 212, Tr 83	Human rib	mature adult	female	26.01	−20.11	9.96	10.84	3.0	2	*
Ostojićevo	Grave 213, Tr 85	Human rib	mature adult	female	41.29	−19.53	16.16	11.28	3.0	2	
Ostojićevo	Grave 218, Tr 84	Human rib	neonate	subadult	40.09	−18.74	15.64	14.26	3.0	2	
Ostojićevo	Grave 219, Tr 84	Human rib	juvenile A	subadult	43.55	−19.38	17.03	10.71	3.0	2	
Ostojićevo	Grave 224, Tr 91	Human rib	mature adult	female	33.95	−19.74	13.07	11.57	3.0	2	
Ostojićevo	Grave 226, Tr 91	Human rib	adult	male	31.24	−19.21	11.93	11.37	3.1	1	
Ostojićevo	Grave 227, Tr 88	Human rib	juvenile A	subadult	6.20	−18.71	2.04	10.82	3.6	2	*
Ostojićevo	Grave 228, Tr 88	Human rib	juvenile b	subadult	26.37	−19.07	9.92	10.95	3.1	1	*
Ostojićevo	Grave 230, Tr 82	Human rib	younger adult II	male	30.09	−18.87	11.37	11.91	3.1	1	
Ostojićevo	Grave 231, Tr 90	Human rib	juvenile b	subadult	21.78	−19.37	8.09	10.68	3.1	2	*
Ostojićevo	Grave 233, Tr 92	Human rib	older adult	male	37.80	−19.50	15.51	11.28	2.8	2	
Ostojićevo	Grave 235, Tr 96	Human rib	younger adult I	male	10.73	−19.42	3.94	11.62	3.2	1	*
Ostojićevo	Grave 240, Tr 102	Human rib	neonate	subadult	39.25	−19.25	16.05	12.59	2.9	2	
Ostojićevo	Grave 246, Tr 96	Human rib	older adult	female	32.15	−19.40	13.14	10.83	2.9	2	
Ostojićevo	Grave 248, Tr 104	Human rib	infant IB	subadult	37.95	−19.05	15.54	12.96	2.9	2	
Ostojićevo	Grave 258, Tr 106	Human rib	older adult	female	20.87	−19.72	8.23	10.79	3	2	*
Ostojićevo	Grave 260, Tr 111	Human rib	younger adult I	male	24.38	−19.40	9.79	11.67	2.9	2	*
Ostojićevo	*Grave 263, Tr 110*	*Human rib*	*mature adult*	*female*	0.00	−79.59	0.09	29.61	*0*	*2*	*
Ostojićevo	Grave 264, Tr 113	Human rib	older adult	female	20.85	−19.82	8.28	11.81	2.9	2	*
Ostojićevo	Grave 266, Tr 115	Human rib	mature adult	male	36.31	−19.79	13.98	10.58	3.0	2	
Ostojićevo	Grave 269, Tr 120	Human rib	older adult	male	29.98	−19.38	11.54	11.11	3.0	1	
Ostojićevo	Grave 270, Tr 119	Human rib	younger adult I	male	18.29	−18.98	6.79	11.09	3.1	2	*
Ostojićevo	Grave 273, Tr 125	Human rib	mature adult	male	13.99	−18.94	5.15	10.92	3.2	2	*
Ostojićevo	Grave 275, Tr 121	Human rib	juvenile A	subadult	31.69	−19.10	12.40	9.70	3.0	2	
Ostojićevo	Grave 28, Trench 10	Human rib	younger adult II	female	42.20	−19.61	15.62	11.23	3.2	2	
Ostojićevo	Grave 281, Tr 135	Human rib	child I	subadult	34.54	−19.35	13.70	10.97	2.9	1	
Ostojićevo	Grave 282, Tr 135	Human rib	infant IA	subadult	38.62	−19.10	15.26	14.12	3.0	1	
Ostojićevo	Grave 29, Trench 11	Human rib	younger adult I	female	42.72	−19.93	15.87	11.46	3.1	2	
Ostojićevo	Grave 30, Trench 11	Human rib	younger adult I	female	42.15	−20.27	15.65	10.01	3.1	2	
Ostojićevo	Grave 34, Trench 12	Human rib	mature adult	male	42.88	−19.93	16.03	12.05	3.1	2	
Ostojićevo	Grave 39, Trench 14	Human rib	neonate	subadult	18.73	−18.73	7.02	14.41	3.1	2	*
Ostojićevo	Grave 41, Trench 14	Human rib	younger adult II	female	43.39	−19.74	16.07	10.74	3.2	2	
Ostojićevo	Grave 42, Trench 14	Human rib	older adult	female	38.34	−19.95	14.27	11.58	3.1	2	
Ostojićevo	Grave 53, Trench 19	Human rib	mature adult	male	41.25	−19.62	15.58	11.20	3.1	2	
Ostojićevo	Grave 59, Trench 20	Human rib	younger adult II	male	39.98	−19.73	15.22	11.25	3.1	2	
Ostojićevo	Grave 6, Trench 5	Human rib	older adult	female	42.24	−19.83	15.43	11.57	3.2	2	
Ostojićevo	Grave 63, Trench 23	Human rib	older adult	male	24.27	−20.01	9.29	10.19	3.0	2	*
Ostojićevo	Grave 64, Trench 23	Human rib	mature adult	female	40.88	−20.01	15.53	11.82	3.1	2	
Ostojićevo	Grave 66B, Tr 22	Human rib	younger adult II	male	29.93	−19.74	11.04	11.49	3.2	2	
Ostojićevo	Grave 67, Trench 23	Human rib	older adult	male	43.89	−19.35	16.39	10.90	3.1	2	
Ostojićevo	Grave 68, Trench 22	Human rib	older adult	female	39.75	−19.66	15.14	10.87	3.1	2	
Ostojićevo	Grave 69, Trench 22	Human rib	younger adult I	female	30.64	−19.86	11.47	9.88	3.1	1	
Ostojićevo	Grave 78, Trench 24	Human rib	younger adult II	female	41.22	−19.21	15.69	10.83	3.1	1	
Ostojićevo	Grave 79, Trench 24	Human rib	juvenile b	subadult	26.08	−19.25	9.72	10.92	3.1	1	*
Ostojićevo	Grave 81, Trench 24	Human rib	older adult	female	43.61	−19.74	16.55	11.53	3.1	1	
Ostojićevo	Grave 82, Trench 24	Human rib	younger adult II	female	36.46	−19.32	13.84	11.43	3.1	1	
Ostojićevo	Grave 86, Trench 24	Human rib	younger adult II	male	14.87	−20.28	5.26	12.21	3.3	2	*
Ostojićevo	Grave 87, Trench 27	Human rib	infant IA	subadult	44.01	−18.92	16.50	11.92	3.1	1	
Ostojićevo	Grave 92, Trench 30	Human rib	younger adult II	female	40.19	−18.89	15.17	11.12	3.1	2	
Ostojićevo	Grave 94, Trench 30	Human rib	younger adult II	male	41.89	−19.61	15.92	11.04	3.1	1	
Ostojićevo	Grave 96, Trench 29	Human rib	mature adult	female	43.10	−19.51	16.42	10.04	3.1	2	
Ostojićevo	Grave 98, Trench 30	Human rib	mature adult	female	39.69	−19.65	15.37	11.19	3.0	2	
Mokrin	Grave 209, Tr 56	Human fibula	mature adult	female	39.11	−19.33	15.10	10.63	3.0	1	
Mokrin	Grave 217	Human rib	younger adult II	male	41.78	−19.16	16.09	10.27	3.0	1	
Mokrin	Grave 222, Tr 58	Human rib	mature adult	female	43.87	−19.15	17.39	10.78	2.9	1	
Mokrin	Grave 232, Tr 59	Human rib	younger adult I	male	43.01	−19.06	16.97	11.01	3.0	1	
Mokrin	Grave 238, Tr 63	Human rib	juvenile	subadult	26.71	−19.59	9.90	10.43	3.1	1	*
Mokrin	Grave 240, Tr 63	Human rib	older adult	male	22.92	−19.37	8.50	10.17	3.1	1	*
Mokrin	Grave 243, Tr 64	Human rib	younger adult II	male	18.05	−19.01	6.72	11.33	3.1	1	*
Mokrin	Grave 246, Tr 66	Human rib	mature adult	female	8.72	−19.24	3.02	10.47	3.4	1	*
Mokrin	Grave 247, Tr 66	Human rib	child II	subadult	40.44	−18.87	15.51	10.18	3.0	1	
Mokrin	Grave 248, Tr 66	Human fibula	mature adult	female	26.37	−19.38	9.91	12.00	3.1	1	*
Mokrin	Grave 249	Human rib	older adult	male	41.52	−19.15	15.89	11.01	3.0	1	
Mokrin	Grave 251	Human rib	mature adult	male	15.93	−19.14	5.72	10.86	3.2	1	*
Mokrin	Grave 253	Human rib	younger adult II	male	23.99	−19.38	8.97	10.87	3.1	1	*
Mokrin	Grave 259, Tr 68	Human rib	younger adult II	male	37.09	−19.11	13.59	10.73	3.2	1	
Mokrin	Grave 260	Human rib	juvenile	subadult	35.42	−19.11	13.16	9.63	3.1	1	
Mokrin	Grave 261, Tr 68	Human rib	younger adult I	female	10.41	−18.87	3.42	10.16	3.6	1	*
Mokrin	Grave 270	Human rib	younger adult II	female	10.30	−19.39	3.36	10.87	3.6	1	*
Mokrin	Grave 273	Human rib	mature adult	male	41.90	−19.40	15.60	10.12	3.1	1	
Mokrin	Grave 279, Tr 73	Human rib	younger adult II	female	15.86	−19.40	5.38	10.58	3.4	1	*
Mokrin	Grave 281, Tr 74	Human tibia	older adult	male	15.81	−19.16	5.47	11.18	3.4	1	*
Mokrin	Grave 287, Tr 76	Human rib	younger adult II	female	30.18	−19.35	10.91	10.90	3.2	1	
Mokrin	Grave 290, Tr 77	Human rib	infant II	subadult	34.27	−19.75	12.39	10.59	3.2	1	
Mokrin	Grave 293, Tr 78	Human rib	younger adult I	female	16.83	−19.51	5.74	11.02	3.4	1	*
Mokrin	Grave 50	Human rib	younger adult I	male	36.90	−19.30	14.12	9.71	3.0	1	
Mokrin	Grave 69, Tr 21	Human rib	younger adult I	female	40.47	−19.17	15.72	10.19	3.0	1	
Mokrin	Grave 81	Human femur	mature adult	male	36.78	−19.15	14.16	11.01	3.0	1	
Mokrin	Grave 84, Tr 24	Human femur	older adult	female	41.42	−19.18	16.36	11.22	3.0	1	
Mokrin	Grave 88	Human femur	younger adult I	female	41.67	−19.31	16.25	10.89	3.0	1	
Mokrin	Grave 92, Tr 28	Human femur	mature adult	male	43.12	−19.18	16.84	10.70	3.0	1	

Faunal remains (see Tables 2 and 3) included specimens of several taxa included as grave offerings at Ostojićevo, Serbia (*n* = 7) as well as faunal specimens uncovered during settlement excavations at Kiszombor-Új-Élet (2700−1950 BCE), an Early Moriš site in southeastern Hungary (*n* = 9), and Klárafalva-Hajdova (2300−1500 cal BCE), a primarily Late Moriš fortified tell site in southeastern Hungary (*n* = 7) [28,11,8]. A total of 23 faunal specimens representing six taxa were sampled: *Bos taurus* (domestic cattle), *Sus scrofa* (domestic pig), *Caprinae* (sheep/goat), *Equus caballus* (domestic horse), *Canis familiaris* (domestic dog), and *Cervus elaphus* (red deer) (Table 2). Faunal samples were obtained with permission from the Narodni Muzej Kikinda, Serbia, for Ostojićevo and the Móra Ferenc Museum, Szeged, Hungary for Kiszombor-Új-Élet, and Klárafalva-Hajdova (Table 4). No permits were required for the described study, which complied with all relevant regulations.

**Table 2:** Stable carbon and nitrogen isotope values of fauna.

Site	Context	Species	Element	%C	δ13C	% N	δ15N	C:N	Phase
Ostojićevo	Grave 101, Tr 28	*Equus caballus*	Astragalus	38.55	−19.99	15.47	7.29	2.5	/
Ostojićevo	Grave 106, Tr 31	*Ovis aries*	Humerus	38.29	−20.18	15.25	7.88	2.5	1
Ostojićevo	Grave 131, Tr 43	*Bos taurus*	Scapula	41.04	−19.57	16.59	7.85	2.5	/
Ostojićevo	Grave 131, Tr 43	*Ovis aries*	Humerus	40.89	−18.05	16.28	7.92	2.4	/
Ostojićevo	Grave 131, Tr 43	*Bos taurus*	Rib	40.04	−18.35	16.50	7.95	2.5	/
Ostojićevo	Grave 14, Tr 7	*Canis familiaris*	3rd metatarsal	37.98	−20.63	15.41	9.76	2.5	/
Ostojićevo	Grave 209, Tr 83	*Ovis aries*	Femur	40.82	−17.34	16.27	9.77	2.5	2
Ostojićevo	Grave 270, Tr 119	*Ovis aries*	Humerus	40.09	−17.90	16.07	9.81	2.5	2
Kiszombor Új-Élet	127	*Ovicapridae*	Humerus	46.52	−18.92	17.18	7.65	2.7	2
Kiszombor Új-Élet	183	*Sus scrofa*	Radius	49.51	−20.05	18.32	7.51	2.7	1
Kiszombor Új-Élet	309	*Sus scrofa*	Ulna	46.58	−20.60	17.13	9.01	2.7	1
Kiszombor Új-Élet	321	*Ovicapridae*	Metatarsel	48.79	−19.62	18.14	6.11	2.7	1
Kiszombor Új-Élet	349	*Bos taurus*	Cow metapodial	49.29	−19.86	18.09	7.41	2.7	1
Kiszombor Új-Élet	409	*Equus caballus*	Scapula	44.18	−21.03	16.32	4.71	2.7	1
Kiszombor Új-Élet	491	*Bos taurus*	Cow metapodial	45.20	−18.82	16.48	7.58	2.7	1
Kiszombor Új-Élet	572	*Cervus elaphus*	Ulna	45.97	−20.32	17.02	6.62	2.7	1
Kiszombor Új-Élet	622	*Bos taurus*	Cow tibia	48.76	−19.84	18.11	9.16	2.7	1
Klárafalva Hajdova	91	*Sus scrofa*	Humerus	48.08	−21.09	18.19	9.41	2.6	2
Klárafalva Hajdova	100	*Sus scrofa*	Phalange	46.92	−20.33	17.54	7.23	2.7	2
Klárafalva Hajdova	221	*Bos taurus*	Phalange	48.55	−21.17	18.28	8.01	2.7	2
Klárafalva Hajdova	269	*Bos taurus*	Humerus	47.87	−19.56	17.95	6.65	2.7	2
Klárafalva Hajdova	400	*Ovicapridae*	Radius	48.28	−18.99	18.34	7.95	2.6	2
Klárafalva Hajdova	733	*Ovicapridae*	Ulna	45.56	−21.25	17.24	7.28	2.6	2
Klárafalva Hajdova	737	*Cervus elaphus*	Phalange	47.95	−20.99	18.13	10.47	2.6	2

**Table 3:** Comparison of stable carbon and nitrogen values among ruminant livestock (cattle, horse, sheep, goat) separated by site.

Herbivores	Carbon					Nitrogen				
	Minimum	Maximum	Average	Range	Standard Dev	Minimum	Maximum	Average	Range	Standard Dev
Ostojićevo	−20.2	−17.3	−18.8	2.8	1.0	7.3	9.8	8.4	2.5	0.9
Kiszombor-Új-Élet	−21.0	−18.8	−19.7	2.2	0.7	4.7	9.2	7.1	4.5	1.4
Klárafalva Hajdova	−21.3	−19.0	−20.2	2.3	1.0	6.7	8.0	7.5	1.4	0.6

**Table 4:** Comparison of stable carbon and nitrogen values among adults and subadults (separated by cemetery and phase).

Human	Carbon					Nitrogen				
	Minimum	Maximum	Average	Range	Standard Dev	Minimum	Maximum	Average	Range	Standard Dev
Ostojićevo Phase 1 Adults	−20.2	−18.9	−19.4	1.3	0.4	9.9	12.8	11.3	2.9	0.7
Ostojićevo Phase 1 Subadults	−20.9	−18.9	−19.4	2.0	0.6	9.0	14.1	11.6	5.1	1.4
Ostojićevo Phase 2 adults	−20.3	−17.2	−19.6	3.1	0.5	9.6	12.1	11.1	2.4	0.6
Ostojićevo Phase 2 subadults	−20.5	−18.5	−19.4	2.0	0.5	9.7	15.2	12.5	5.5	1.5
Mokrin adults	−19.4	−19.1	−19.2	0.3	0.1	9.7	11.2	10.7	1.5	0.4
Mokrin subadults	−19.7	−18.9	−19.2	0.9	0.5	9.6	10.6	10.1	1.0	0.5

Human (rib where possible, and femur, tibia, or fibula) and animal bones (metatarsals, astragalus, scapula, humerus, rib, femur, radius, ulna, phalanges, metapodial) were sampled with a Dremel® Micro 8050 drill using a 545-diamond cutting wheel attachment. Laboratory analysis was carried out at the Leibniz Laboratory for Radiometric Dating and Stable Isotope Research at the University of Kiel. Bone samples were demineralized in 0.5 M EDTA (pH 8.0) with a change of acid every other day until collagen was translucent and flexible. The resulting collagen pseudomorphs were rinsed in distilled water seven times and then soaked overnight to remove residual EDTA solution. After this, they were rinsed a further eight times. Samples were then placed in 0.1 NaOH to remove humic acids and further rinsed five times in distilled water. The collagen samples were then freeze-dried and weighed for analysis. Stable carbon (δ^13^C) and nitrogen (δ^15^N) isotope analysis was undertaken at the Boston University Stable Isotope Laboratory using a EuroVector Euro EA elemental analyser coupled with a GVI IsoPrime in continuous flow mode. Analytical error was 0.1‰ and 0.2‰ for δ^13^C and δ^15^N, respectively. Isotopic values are reported in permil (‰) relative to the Vienna Pee Dee Belemnite (VPDB) standard for δ^13^C and atmospheric nitrogen (AIR) for δ^15^N. Samples with a carbon-to-nitrogen (C/N) ratio outside the range of 2.9 to 3.6 were excluded for potential contamination and/or insufficient preservation of biogenic carbon or nitrogen [70]. Additionally, samples displaying wt. %C below 30% and/or wt. %N below 11% were also excluded, even if the associated C:N value was within the normal range as they could indicate insufficient organic content, contamination or degradation or other analytical precision problems [71].

### Isotopic baselines

Variations in δ^13^C_collagen_ in temperate terrestrial ecosystems, especially in animals, can reflect local climatic and geographic conditions (see Supplement) as well as species-specific foraging patterns and preferences for C_3_ plants versus C_4_ grasses and sedges [55,72]. Floral biomass in the Pannonian Plain includes a mix of grasses and flowering woody C_3_ plants, trees, and shrubs, alongside some C_4_ grasses and Chenopodiaceae [Deak, 2005,^73^]. ^13^C enrichment in C_3_ plants is due to increases in water-use efficiency associated with a decrease in water availability due to high soil salinity (leaves, stems, and roots) and high solar irradiance (leaves) [74,75]. In contrast, C_4_ plants exhibit little correlation between δ^13^C values and water availability [74]. The primary factor influencing the δ^15^N values of plants are source nitrogen and the process by which plants uptake soil nitrogen pools (*i.e.*, direct or mediated by symbiotic microbes) [76]. Soil and by extension plant δ^15^N values are further impacted by annual precipitation, soil salinity, and manure from animals grazing in pastures or applied as fertilizer to agricultural plots [59,77–79].

## Isotopic results

**Fig 3:**
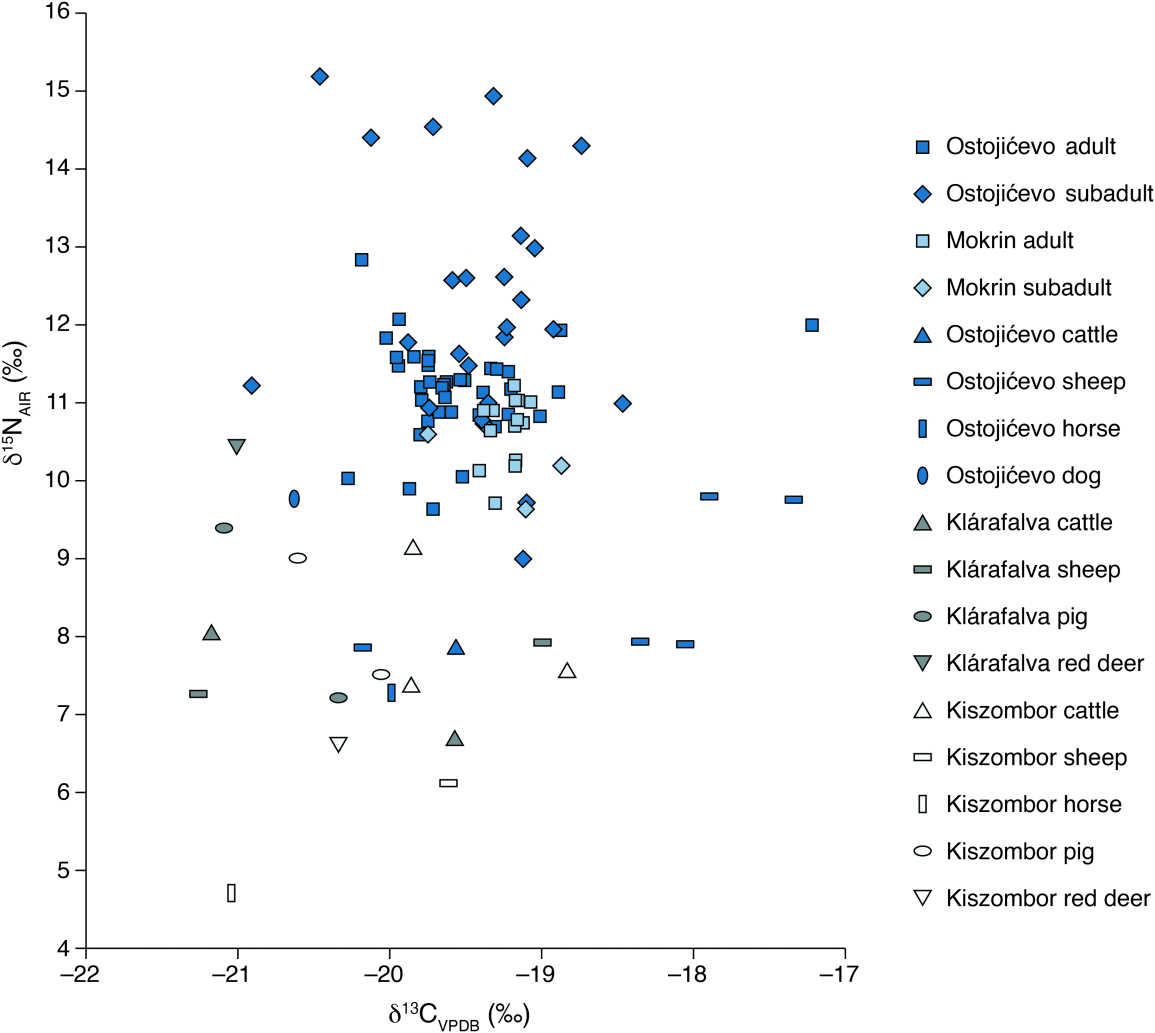
Stable carbon and nitrogen isotopes of humans and fauna from all sites.

Here, we present the results of stable carbon (δ^13^C) and nitrogen (δ^15^N) isotopic analyses of human and faunal remains from the cemetery of Ostojićevo, as well as human remains from the cemetery of Mokrin. In addition, we analyzed fauna from settlements of Kiszombor-Új-Élet and Klárafalva-Hajdova in an effort to bolster available reference data from the region and time period of study (Tables 1 through 2; Figs 3 through 6).

**Fig 4:**
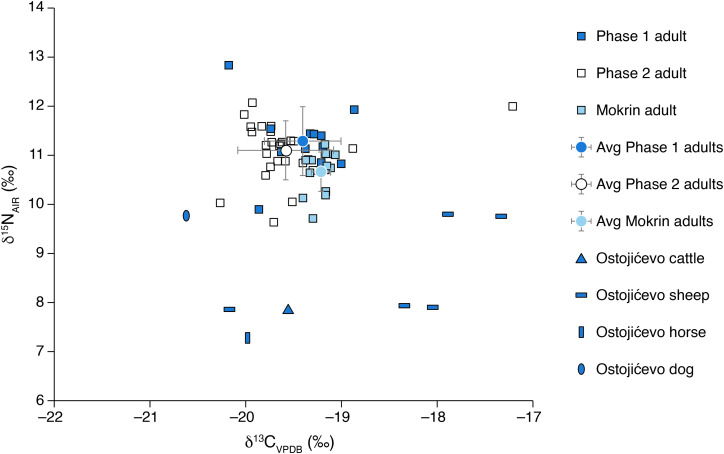
Stable carbon and nitrogen isotopes of adult individuals from Ostojićevo (Phases 1 and 2) and Mokrin.

**Fig 5:**
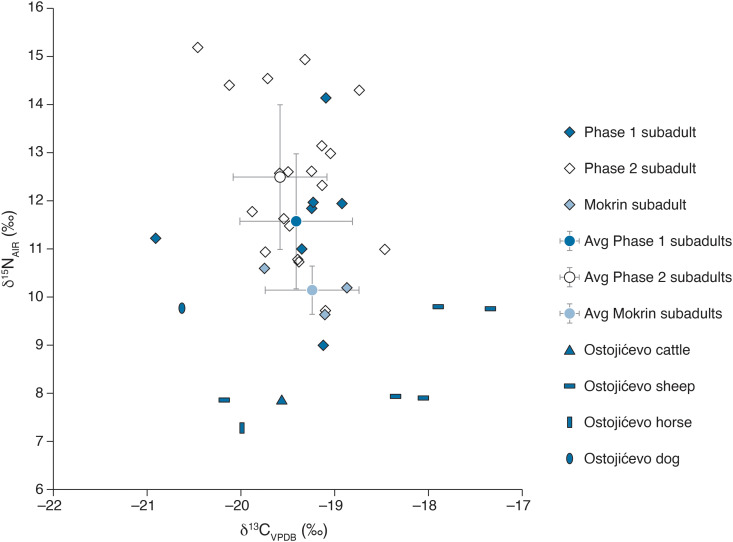
Stable carbon and nitrogen isotopes of subadult individuals from Ostojićevo (Phase 1 and 2) and Mokrin.

**Fig 6:**
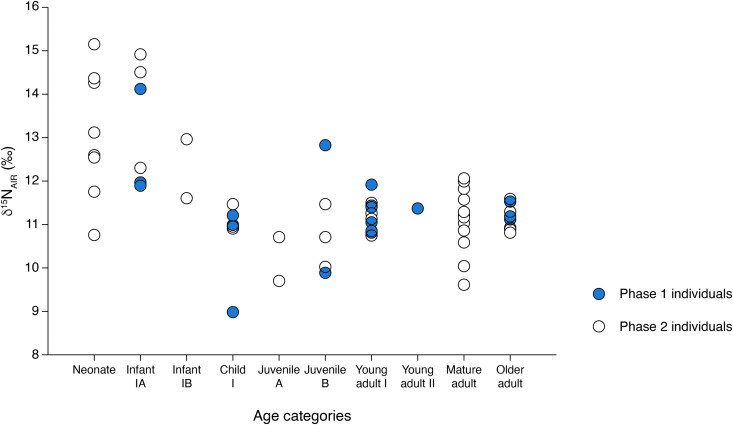
Stable nitrogen isotope values of individuals across age categories at Ostojićevo.

### Ostojićevo

Of 89 original samples, 24 specimens (26.9%) did not meet the criteria for collagen preservation (see methods) and were excluded from analysis (Table 1). After removal of samples, the resulting sample set included 26 subadults and 39 adults. Carbon isotope values of humans at Ostojićevo ranged from −20.9 to −17.2‰, with an average of −19.5 ± 0.5‰ (Figs 4a, 4b). Adults had δ^13^C values that ranged from −20.3 to −18.9‰, except for a single individual with a value of −17.2‰. δ^13^C values of subadult individuals ranged from −20.9 to −18.5‰. The average δ^13^C value of adults was −19.5‰, while for subadults it was −19.4‰.

The Ostojićevo isotopic dataset was subsequently divided into two phases (pre- and post-1850 BCE) to reflect cultural shifts in cemetery use over time that may correspond to broader social and/or economic changes. Adult δ^13^C values range from −20.3 to −18.9‰ (phase 1) and −20.3 to −17.2‰ (phase 2). Two sample T-tests comparing the δ^13^C values revealed that at 95% confidence there were no significant differences between adults from phases 1 and 2 (df = 37; t Stat = 1.03, p = 0.31), nor were there significant differences between subadults (df = 24; t Stat = 0.04, p = 0.97). There was much greater variation in stable nitrogen isotope values between adults and subadults, as well as among subadult groupings (Figs 3–5). Stable nitrogen isotope values ranged from 9.0 to 15.2‰, with subadults having a greater range of values (9.0 to 15.2‰) compared to adults (9.6 to 12.8‰). Among subadults, neonates and infants had the highest values ranging from 10.7 to 15.2‰, while children and adolescents had δ^15^N values from 8.9 to 11.8‰ (Fig 6).

As discussed above, we split the isotopic data from Ostojićevo into two phases, with adult δ^15^N values ranging from 9.9 to 12.8‰ (phase 1) and 9.6 to 12.1‰ (phase 2). T-tests comparing the δ^15^N values reveal that at 95% confidence there were no significant differences between adults from phases 1 and 2 (df = 37; t Stat = 0.88, p = 0.39), nor were there significant differences between subadults (df = 24; t Stat = −1.32, p = 0.20).

### Mokrin

Of 29 original samples, 12 (41.4%) did not meet criteria for collagen preservation and were excluded from analysis (see methods). The adjusted sample (n = 17) included three subadults and fifteen adults. Stable carbon isotope (δ^13^C) values of adults were tightly clustered, with a range from −19.4 to −19.1‰ (Fig 4). Similarly, subadults exhibited minimal variation in δ^13^C values, with a range from −19.8 to −18.9‰ (Fig 5). There was only slightly more variation in stable nitrogen isotope (δ^15^N) values, with adults ranging from 9.7 to 11.2‰ compared to subadults whose values ranged from 9.6 to 12.8‰. Among subadults, an infant had the highest value of 10.6‰, while children and adolescents had δ^15^N values from 9.6 to 10.2‰.

Two sample T-tests comparing the δ^13^C values from Mokrin and Ostojićevo (phase 1) reveal that at 95% confidence there were no significant differences between adults (df = 24; t Stat = −1.84, p = 0.08), nor were there significant differences between subadults (df = 8; t Stat = −0.39, p = 0.71). Two sample T-tests comparing the δ^15^N values from Mokrin and Ostojićevo (phase 1) reveal that at 95% confidence there were significant differences between adults (df = 24; t Stat = 2.78, p = 0.01), but that there were not significant differences between subadults (df = 8; t Stat = 1.54, p = 0.16).

### Isotopes of fauna

A total of 24 faunal specimens were sampled from the sites of Ostojićevo (n = 8), Kiszombor-Új-Élet (n = 9), Klárafalva-Hajdova (n = 7), with samples yielding C/N ratios of 2.9 to 3.2. These included livestock, such as cattle, sheep, goats, and horses, as well as pigs, red deer, and a dog. Animals from different archaeological sites were found to vary isotopically. At the site of Ostojićevo, sheep had δ^13^C values ranging from −20.2 to −17.3‰, the cattle had values ranging from −19.6 to −18.1‰, while the horse had a value of −20‰ and a dog had a value of −20.6‰. Comparatively, ruminants from Kiszombor-Új-Élet (n = 5) had δ^13^C values ranging from −19.9 to −18.8‰, with the horse having a value of −21.0‰. Pigs at Kiszombor-Új-Élet (n = 2) had δ^13^C values of −20.1 and −20.6‰, and a single red deer with a value of −20.3‰. Finally, at Klárafalva-Hajdova, ruminants (n = 4) had δ^13^C values ranging from −21.3 to −19.0‰, while pigs had values of −21.1 and −20.3‰, respectively, and a red deer had a value of −21.0‰. T-tests comparing the δ^13^C values of livestock (cattle, horses, sheep, goats) reveal that at 95% confidence there were no significant differences between the sites (Ostojićevo / Kiszombor: df = 11; t Stat = 0.17, p = 0.13; Ostojićevo / Klarafalva: df = 9; t Stat = 2.08, p = 0.07; Kiszombor / Klarafalva: df = 8; t Stat = 0.92, p = 0.39). Although, isotopic variation was more evident between Ostojićevo and Klarafalva (p = 0.07), than for other sites.

Faunal δ^15^N values also varied widely, ranging from 4.7 to 10.5‰. Comparatively, at Kiszombor, both ruminants and pigs had similar ranges of δ^15^N values from 6.1 to 9.2‰ and 7.5 to 9.0‰, respectively. The lowest relative δ^15^N values were of red deer (6.6‰) and horses (4.7‰). At the site of Klárafalva-Hajdova, red deer had the highest δ^15^N values of 10.5‰, pigs had values of 7.2 and 9.4‰, while ruminants had δ^15^N values ranging from 6.6 to 8.0‰. Two sample T-tests comparing the δ^15^N values of livestock (cattle, horses, sheep, goats) reveal that at 95% confidence there were no significant differences between the sites (Ostojićevo / Kiszombor: df = 11; t Stat = 1.77, p = 0.10; Ostojićevo / Klarafalva: df = 9; t Stat = 1.56, p = 0.15; Kiszombor / Klarafalva: df = 8; t Stat = −0.45, p = 0.66).

## Discussion

The combined date range for the Moriš cemeteries of Mokrin and Ostojićevo is 2100–1500 cal BCE, which is divided in this study into two phases (Phase 1, 2100−1850 BCE and Phase 2, 1850−1550 BCE) based on mortuary evidence and radiocarbon dates. The cemetery of Mokrin falls within Phase 1, while the cemetery of Ostojićevo has burials dating to both phases. Archaeological evidence suggests that over this 550 year period there was a shift in Moriš mortuary traditions towards fewer and less elaborate grave offerings and the inclusion of neonates and infants in community cemeteries, yet the δ^13^C and δ^15^N isotope values measured in bone collagen of individuals from Ostojićevo and Mokrin do not indicate a change in diet. Thus, the sociocultural and economic forces driving shifts in mortuary customs did not affect subsistence, which was stable over a long period of time. It is unclear whether the inclusion of neonates and infants reflects an increase in infant mortality in the later phase, as mortality patterns among other age cohorts appear to remain stable over time [16]. Regardless, the communities who buried their dead at Mokrin and Ostojićevo seemed to have exploited similar subsistence strategies over both space and time, especially as these relate to key sources of dietary protein.

Isotopic reference data for the sites under study is sparse, with a lack of isotopic values for plants from the southern Carpathian Basin region. While Ostojićevo had limited fauna available for analysis, which were included as grave offerings or incidental finds intermixed with human remains, the site of Mokrin lacked fauna available for sampling. Thus, samples were collected from two Moriš settlement sites (Kiszombor-Új-Élet and Klárafalva-Hajdova) with robust and well-documented faunal assemblages that temporally overlap with the two cemeteries included in this study. It is unclear where those interred at Mokrin and Ostojićevo lived, since Moriš cemeteries are separate from settlements, and the size and duration of use of individual cemeteries indicate their simultaneous utilization by several villages or hamlets. Faunal isotope values exhibit wide variation, with ruminants from Kiszombor-Új-Élet and Klárafalva-Hajdova having lower values than those at Ostojićevo. These settlement sites are located to the north of the cemeteries, closer to the confluence of the Tisza and Maros rivers. The cemetery of Ostojićevo was located directly next to the Tisza river, while Mokrin was further inland. It is likely that the two settlements were exposed to seasonal river flooding and had prominent wetland environments.

This variation in the micro-environmental conditions associated with the respective site locations is evident isotopically, with ruminant livestock from Ostojićevo ingesting a greater proportion of C_4_ plants than those at the two settlements to the north. Similarly, the horse at Ostojićevo had a much higher nitrogen value (7.3‰) than the one at Kiszombor-Új-Élet (4.7‰). Due to the small sample size, lack of information on where those interred at Ostojićevo lived, and limited data on mobility within and beyond the Moriš culture area, it is unclear whether this variation was due to microenvironmental differences or grazing behavior. Red deer are present in the assemblages at Kiszombor-Új-Élet and Klárafalva-Hajdova, but are absent from Ostojićevo; however, faunal materials at Ostojićevo are more likely to reflect deliberate ritual practices (e.g., food consumption around the time of the burial event, deliberate funerary offerings, etc.) rather than byproducts of daily life. Nevertheless, there is strong isotopic evidence from fauna at Ostojićevo that corresponding to drier landscapes or a greater presence of C_4_ vegetation which can be compared to Kiszombor-Új-Élet and Klárafalva-Hajdova, which have isotopic evidence for more C_3_ vegetation suggesting either wetter landscapes or a forested area. These differences in the isotopic values of fauna from the two settlement sites to the north relative to Ostojićevo underscores their lack of utility as comparative reference data for Ostojićevo or Mokrin. However, the faunal data from Kiszombor-Új-Élet and Klárafalva-Hajdova are informative in that they enable broader regional comparisons of the dynamic interplay between Moriš foodways and micro-environments. Overall, isotopic variation between fauna highlights the need to explore intra-site variability, which warrants further research.

At Ostojićevo, adults from phases 1 and 2 had average δ^13^C values of −19.4 and −19.6‰, respectively, but there were no significant differences between these groups (as discussed in the results). However, one individual from the second phase had a δ^13^C value of −17.2‰, which was an outlier compared to all other individuals. It is possible that this individual lived during the very end of the late phase or could have arrived from another location, as their carbon isotope value varies from the rest of the phase 2 group. As the human δ^13^C and δ^15^N values do not vary significantly by phase, these analyses indicate that dietary intake was relatively stable over a prolonged period of time. Thus, while there were distinct differences in material culture, for example an increased intensity of horses and metallurgy from the Early and Late Moriš, there was not a corollary change in human subsistence patterning.

Overall, at Ostojićevo, the average δ^13^C values of adults (−19.5‰) was depleted relative to livestock (−18.8‰), with nitrogen values for adult humans (11.2‰) that were within a few per mil of ruminants (8.5‰). This suggests that human dietary intake was focused primarily on domesticated livestock, mainly ruminants. It could be that the community was also consuming wild foods that were hunted or foraged, as well as domestic C_3_ plants. While diets at Ostojićevo may have included domesticated plants, such as wheat or barley, these do not seem to have been an important part of dietary intake during these periods.

The dietary intake of individuals from the site of Mokrin was more challenging to determine, as no faunal remains were sampled from this location. Faunal remains from Ostojićevo were selected as the most representative reference dataset to compare the Mokrin results, as it was the closest site with available data and the Mokrin cemetery was still active during Ostojićevo phase 1 (Figs 3 and 4). The average δ^13^C values of adults from Mokrin were tightly clustered at −19.2‰. Comparatively, reference values from fauna from Ostojićevo include livestock with an average value of −18.8‰. Among adults at Mokrin and Ostojićevo (phase 1) there was a significant difference in nitrogen isotope values; however, this 0.7‰ variation might be explained by the greater number of young adults at Ostojićevo (75%) compared to Mokrin (50%). In terms of carbon isotopes, the adults at the two sites (Mokrin and Ostojićevo, phase 1) were not significantly different. Thus, we can confidently interpret adult diets at the site of Mokrin as being focused primarily on pastoral livestock alongside foraging of wild plants and animals, with the potential for a small intake of domesticated C_3_ plants. There was minimal variation between isotope values of adults and subadults at Mokrin, although the latter may be due to much smaller sample sizes for subadults (n = 3).

Dietary intake of subadults did not vary within or between cemeteries, suggesting spatial and temporal stability in subsistence patterns. Isotopic variation between subadults at the sites under study is likely due to differences in the number of neonates, infants, children, and adolescents. For example, during phase 2 of Ostojićevo there were a greater number of neonates and infants than during phase 1 at Ostojićevo or at Mokrin. While there were generally higher δ^15^N values among subadults than adults, as well as among neonates and infants than other subadults, this was to be expected due to the impacts of neonates in the womb as well as the effects of breastfeeding [see 80 for discussion].

## Food stability and diet

The stability of food resources is an essential pillar for the preservation, growth, and development of societies [81,82]. A surplus of food is frequently presented as a key factor driving social change and providing a foundation for increased complexity [82–85]. The resilience of societies is dependent upon available resources, which seem to have changed minimally over the 550 years of Moriš burials that we studied, as represented by a general lack of variation in isotopic values of individuals within or between cemeteries.

During the Early to Late Moriš transition (phase 1, 2100−1850 BCE), which encompasses the site of Mokrin and the early phase of Ostojićevo, dietary intake was very similar. Over roughly three hundred years it seems that, despite intraregional variation in mortuary customs, people engaged in similar subsistence strategies, based primarily on livestock (sheep, goats, cattle, horses) and foraging (Fig 4). The transition to an intensification of horse breeding and increased metallurgy within Moriš settlements to the east corresponded to a consistency in behaviors surrounding subsistence and diet in Moriš communities to the south and southwest. This is supported by the lack of significant dietary changes during the Late Moriš period, as seen at Ostojićevo during the second phase. This suggests an effective long-term management of food production across the southern portion of the Moriš culture area. Overall, there were no significant dietary differences within or between phases or cemeteries, with the Early to Late Moriš transition marked by similarities in diet reflecting stable foodways.

## Conclusion

This manuscript reports the first isotopic data of human and faunal dietary intake for Serbia, resulting in important findings about the stability of resources during the Early and Late Moriš periods. Our inter- and intra-cemetery comparison of dietary trends indicates that Moriš dietary practices did not markedly change over time. Adults at the sites of Mokrin and Ostojićevo had similar diets across the Early to Late Moriš (2100−1550 BCE). Generally, diets were based on consumption of livestock including cattle, sheep, goats, and horses; however, these communities also secondarily consumed wild animals such as red deer, fish, and mollusks, as well as pigs and domestic C_3_ plants. While few botanical specimens have been recovered from these sites, their inclusion in dietary intake is not precluded. While we cannot rule out the consumption of small amounts of domesticated millet at these sites, the translocation of millet into this region probably occurred later in time.

The stability of subsistence resources is an essential component in sustaining societies. Often there are complex links between social systems and subsistence economies, yet in this case we have demonstrated that shifts in mortuary practices and economic practices, notably the degree of horse breeding and metallurgy, in the larger Moriš culture area did not translate to variation in dietary patterns. Moriš societies, at least in the south, built resilient subsistence economies that seem to have changed minimally over 550 years. The presence of infant burials during phase 2 at Ostojićevo raises the possibility of Late Moriš communities facing political unrest or economic stress, especially after 1850 BCE [3,25,27,86–116]. Future research is needed to examine skeletal indicators of stress in age-matched individuals, especially subadults, within and between Moriš cemeteries. Nevertheless, the data presented here suggests cohesion of food management strategies among various Moriš communities over a half millennium, indicating conditions of long-term stability rather than insecurity.

## Supporting information

S1 File Supplemental material including information on Environment and Topography, Climate, and Faunal Analysis. (PDF)
