# Supplementary material for: Dietary stability in ancient Serbia: Isotopic analysis of two middle bronze age Moriš Cemeteries
Source: PLoS One. 2026 Apr 1;21(4):e0344463. doi: 10.1371/journal.pone.0344463 (PMC13042745; doi:10.1371/journal.pone.0344463)
Supplement: S1 File — (PDF) [file pone.0344463.s001.pdf]

S1 Text. Supplemental Material

Dietary Stability in Ancient Serbia: Isotopic Analysis of two Middle Bronze Age Moriš Cemeteries

Iride Tomažič[1,2], Katherine Pompeani[3], Kara Larson[1,2], Amy Nicodemus[4], John O'Shea[1,2], Lidija Milašinović[5], Alicia Ventresca-Miller[1,2]

Affiliations:
1 University of Michigan Museum of Anthropological Archaeology, 2 University of Michigan Department of Anthropology, 3 University of Pittsburgh, 4 University of Wisconsin La Crosse, 5 National Museum of Kikinda Serbia

Environment and Topography
All analyzed sites were located within the Pannonian Plain, characterized by permanent or semi-permanent surface water resulting from the overflow of the Tisza and Moriš Rivers (Balásházy and Somogyi 1989; Kiss and Sipos 2007; Gyucha et al. 2011; Sommerwerk et al. 2009). After the 19th century, the Austro-Hungarian Empire implemented river regulations aiming to decrease the extent of the wetlands and minimize the risk of flooding of the Tisza, Danube, and their tributaries, resulting in a reduction in water levels of the rivers by 3 to 4 meters (Kosse 1979). The highly variable hydrological conditions of the eastern side of the Tisza created the river floodplains, low basins, and wetlands (Kosse 1979; Várallyay and Zilahy 1989; Gulyás and Sümegi 2011). As a result, soil patterns in this region are diverse but are dominated by alluvium and greysols (Deák 2005; Kosse 1979).

Climate
Previous studies aimed to reconstruct the climatic conditions in the Carpathian basin during the past. This was accomplished through the study of pollen cores (Girić 1971; Sümegi and Bodor 2000; Sümegi et al. 2003; Tantau et al. 2005; Nagy-Bodor et al. 2000), water budget, sedimentation reconstruction in lakes and bogs (Tapody et al. 2021; Willis et al. 1995), speleothems (Onac 2002), and macrofaunal, isotopic analysis of shell and animal remains from archaeological sites (Bartosiewicz, 2005; Daróczi-Szabó et al. 2020; Depaermentier et al. 2020; Gulyás and Sümegi 2011; Gyucha et al. 2009; Hoekman-Sites and Giblin 2012).

The majority of past climate reconstructions are based on areas far removed from the area of study, based on pollen cores and sediments in lakes and bogs in the larger Carpathian Basin. According to these studies the Bronze Age (ca. 2700 to 800 BC) was characterized as having a subboreal climate characterized by a wetter and colder environment (Gardner 2002; Nagy-Bodor et al. 2000; Schöll-Barna et al. 2012; Tapody et al. 2021). During the Early (2500-1900 BCE) and Middle Bronze Age (1900-1500 BCE) (Kiss et al. 2019), wetter conditions caused hydrological and ecological changes, resulting in a higher water table, river downcutting, and heavy flooding (Gábris 1998). According to past research, the transition to wetter conditions is especially marked after 2000 BC, with a purported increase in the extent of bogs and marshes (Sherratt 1984; Gyulai 1993). The plains developed a more closed and expanded forest system populated by oak trees and woodland species, especially red deer (Bökönyi 1988). However, the forest expansion was likely offset by extensive deforestation in this period, argued to be caused by metallurgical production (Willis et al. 1995). While these studies give a general idea of the climate in the region, a more detailed version of the climatic conditions within the region is still needed. Some attempts were

made by Girić (1971) who reported pollen data in the vicinity of Mokrin, along with Molloy et al. (2023) who contextualized provided reconstructions of the climate proxy for the southern Pannonian Plain, based upon stable oxygen and hydrogen isotopes and trace element composition of stalagmite from Romanian caves located 200 km west and north form the study region.

Girić (1971:21) presents evidence of a drier and colder climate beginning in 2000 BCE and persisting throughout the entire Bronze Age. In contrast, Molloy et al. (2023:4) provide a more nuanced reconstruction, identifying climatic fluctuations: a cold and wet phase from 2000–1900 BCE, transitioning into a warmer and increasingly arid period between 1900–1700 BCE. After 1700 BCE, the climate appears to have become progressively more unpredictable. While these reconstructions offer valuable insights and present differing perspectives on regional climatic developments, it is crucial to emphasize the need for micro-regional and local climatic reconstructions to refine our understanding of climatic circumstances that could affect social processes further.

Faunal analysis

<u>Fauna from settlements</u>

Faunal analysis from Klárafalva-Hajdova and Kiszombor-Új-Élet was carried out by Nicodemus (for details, refer to Nicodemus 2010; 2014). The types and proportions of domestic and wild animals exploited for food at these two sites reflect different economic strategies linked to their ecological setting. Both sites are similar in that caprines and pigs predominate among domestic taxa. However, there are differences in the relative abundance of domestic taxa and reliance on high-ranking game (Nicodemus 2014). For example, large and small wild game were more abundant at Klárafalva-Hajdova (12%) compared to Kiszombor-Új-Élet, (5%), indicating increased exploitation of forested environments at the former. Overall, greater diversity in the animal economy is documented in the archaeological record for Klárafalva-Hajdova compared to Kiszombor-Új-Élet, with high evidence of fishing and some hunting of high-ranking wild game prioritized during the Early Moriš Period at Klárafalva-Hajdova.

In contrast, the faunal assemblage recovered from Kiszombor-Új-Élet, suggests a more stable animal economy centered on sheep/goat herding, with little to no change in the relative proportion of mammal and fish species exploited over time. Cattle herding was more critical at Kiszombor-Új-Élet than Klárafalva-Hajdova. An increase in the proportion of cattle in the Late Moriš phase at Klárafalva-Hajdova corresponds to a decrease in fishing. In contrast, cattle increase as sheep/goat decline during the Late Moriš occupation at Kiszombor-Új-Élet. This pattern suggests a shift to more grassland-adapted species and higher livestock values between the Early and Late Moriš occupation phases at settlements near the Tisza-Moriš confluence (Nicodemus 2014).

<u>Fauna from cemeteries</u>

Girić (1971) describes the content of each grave from Mokrin and mentions the presence of faunal remains in each grave. Evidence of animal teeth, bones, and mollusks (*Glycymeris, shells of Dentalium, Columbella*) were found either as grave offerings (food remains or bone tools) or ornaments (pendants, beads, clothing pins) at Ostojićevo and Mokrin alike (Vitezović 2001). However, a considerable number of disturbed graves (Morkin 23%, Ostojićevo 16%) obscures the total number of animal remains (Blagojević 2020, 85). Animal offerings were far less common and came from primarily sheep or goats, followed by cattle and pigs, with occasional red deer

(Vitezović 2001). As a result, the faunal assemblage from the cemeteries probably mirrors the herding practices of the Moriš people. However, no evidence for fish, despite its prominence in Klárafalva-Hajdova assemblage, was recovered. This could have been the result of a preservation issue of the fragile bones, or excavation methods, or simply that fish were not part of the mortuary treatment.

Research conducted by Blagojević (2020) presents the animals' remains from both cemeteries and shows differences between the two cemeteries. At Mokrin, animal parts (mandibles, followed by ribs and isolated teeth) were placed around the deceased area and not in bowls. The most common animal species were domestic animals such as cattle, followed by horse, sheep/goat, and pig. In contrast, wild animals were rarer amongst the remains of red deer and Eastern imperial eagle (*Aquila heliaca*). Ostojićevo showed fewer animal offerings in graves in comparison to Mokrin. Animal remains (meat parts, such as humeri, ribs, and vertebrae, and only one grave presented cranial parts) were commonly placed in bowls and less commonly around the individual's head. Individuals were buried with predominantly domestic animals, with the highest number of cattle, followed by pigs, sheep/goat, and dogs. Like Mokrin, Ostojićevo had low numbers of wild animals; the only one identified was a European hare (*Lepus europaeus*) (Blagojević 2020).

All human and animal remains used in this study were from archaeological contexts. We received permission for the collection of samples from the National Museum of Kikinda, Serbia (for Ostojićevo and Mokrin). Samples from Kiszombor-Új-Élet and Klárafalva-Hajdova were provided by A. Nicodemus and J. O'Shea.
